# Effect of neoadjuvant chemoradiation on anorectal function assessed with anorectal manometry: A systematic review and meta-analysis

**DOI:** 10.1177/03008916241256544

**Published:** 2024-05-31

**Authors:** Pamela Milito, Guglielmo Niccolò Piozzi, Mohammad Iqbal Hussain, Tommaso A. Dragani, Luca Sorrentino, Maurizio Cosimelli, Marcello Guaglio, Luigi Battaglia

**Affiliations:** 1 Department of Emergency and General Surgery, IRCCS Policlinico San Donato, Milan, Italy; 2 Colorectal Surgery Unit, Fondazione IRCCS Istituto Nazionale dei Tumori, Milan, Italy; 3 Department of Colorectal Surgery, Portsmouth Hospitals University NHS Trust, Portsmouth, UK; 4 Department of General Surgery, Great Western Hospital NHS Foundation Trust, Swindon, UK; 5 Department of Research, Fondazione IRCCS Istituto Nazionale dei Tumori, Milan, Italy

**Keywords:** Anorectal manometry, LARS, anal function, neoadjuvant chemoradiotherapy

## Abstract

**Aim:**

Improvement in oncological survival for rectal cancer increases attention to anorectal dysfunction. Diagnostic questionnaires can evaluate quality of life but are subjective and dependent on patients’ compliance. Anorectal manometry can objectively assess the continence mechanism and identify functional sphincter weakness and rectal compliance. Neoadjuvant chemoradiotherapy is presumed to affect anorectal function. We aim to assess anorectal function in rectal cancer patients who undergo total mesorectal excision, with or without neoadjuvant chemoradiation, using anorectal manometry measurements.

**Method:**

MEDLINE, Embase, and Cochrane databases were searched for studies comparing perioperative anorectal manometry between neoadjuvant chemoradiation and upfront surgery for rectal cancers. Primary outcomes were resting pressure, squeeze pressure, sensory threshold volume and maximal tolerable volume.

**Results:**

Eight studies were included in the systematic review, of which seven were included for metanalysis. 155 patients (45.3%) had neoadjuvant chemoradiation before definitive surgery, and 187 (54.6%) underwent upfront surgery. Most patients were male (238 vs. 118). The standardized mean difference of mean resting pressure, mean and maximum squeeze pressure, maximum resting pressure, sensory threshold volume, and maximal tolerable volume favored the upfront surgery group but without statistical significance.

**Conclusion:**

Currently available evidence on anorectal manometry protocols failed to show any statistically significant differences in functional outcomes between neoadjuvant chemoradiation and upfront surgery. Further large-scale prospective studies with standardized neoadjuvant chemoradiation and anorectal manometry protocols are needed to validate these findings.

## Introduction

The widespread standardization of total mesorectal excision (TME) and the adoption of multimodal therapy has highly improved the treatment of rectal cancer (RC) with better long-term oncological outcomes.^1^ Technical surgical advancements, with the adoption of low anterior resection (LAR), allow up to 80% of sphincter-saving procedures.^2^ However, up to 80% of patients undergoing LAR will develop a change of bowel habit, with a wide range of symptoms collectively known as low anterior resection syndrome (LARS).^2,3^ LARS is characterized by a combination of fecal and/or gas incontinence, fecal urgency, fragmented defecation, and incomplete evacuation that is significantly associated with a deterioration of Quality of Life (QoL).^2,4-6^ LARS can be considered transient, with symptoms that may resolve after 18 months from surgery. However, there are studies reporting the persistence of symptoms even after 15 years from surgery, with a high burden on patients’ QoL.^2^ Following the improvement of the oncological outcomes, the anorectal function must be carefully considered in the long-term follow-up, however this is still poorly described and investigated in literature, with often poorly performed studies.^7^ Moreover, these studies included heterogenous populations, often with incomplete patient information on baseline, oncological, or functional outcomes.

The etiology of LARS is multifactorial, namely the direct surgical damaging of pelvic/intramural nervous plexuses, the direct damaging of the anal sphincter (through endoanal instrumentation),^8^ the anastomotic technique, the neorectal configuration, and the use of chemoradiotherapy with effects on colonic and neorectal motility.^2^ Radiotherapy and the level of colorectal anastomosis are considered the most consistent factors that negatively impact major LARS.^7,9,10^ Due to the lack of a consensus definition and standardization of LARS, there are various diagnostic tools in the form of questionnaires.^11-18^ The most commonly used are the LARS score,^13^ the Wexner score,^14^ and the Memorial Sloan Kettering Cancer Center Bowel Function Instrument.^12^ The great disadvantage of these questionnaires comes from their subjective nature and patients’ compliance in referring their QoL. Anorectal manometry (ARM) measures the pressure in the rectum/anal canal through a pressure transducer.^19^ ARM can objectively assess the quality of the continence mechanism, as it can identify functional sphincter weakness and rectal compliance and can be used to evaluate alternative procedures to stoma.^20,21^ However, ARM technology (i.e., type of probes), methodology and protocols (i.e., timing of execution), and investigated parameters are not yet standardized in the literature.

Until now, none of the available diagnostic questionnaires can provide a complete and comprehensive assessment of the complexity of the anorectal function after LAR with further attention to neoadjuvant chemoradiation (nCRT). This study aims to explore the role of ARM as a diagnostic tool for functional anorectal disorders in patients treated with LAR with or without nCRT.

## Methods

### Literature search

The study was conducted according to the Preferred Reporting Items for Systematic Reviews and Meta-analyses (PRISMA) statement.^22^ Literature was reviewed from 2000 to 2024 using PubMed, MEDLINE, Embase, and Cochrane databases to identify English-language studies.

The search terms were “rectal cancer”, “manometry” and “radiotherapy”. The reference lists of each article were consulted for additional studies. Two authors (PM and MIH) edited a list of studies, meeting the inclusion criteria, by screening titles and abstracts. Three authors (PM, LB and MIH) independently extracted the data from the eligible studies. The latest date for this search was 28 March 2024. The study was registered on PROSPERO (The International Prospective Register of Systematic Reviews) accessible at www.crd.york.ac.uk/prospero (Registration number: CRD42021232302).

### Eligibility criteria and data extraction

All retrospective and prospective studies of RC treated with LAR who underwent anorectal manometry examination before and after surgery were eligible for inclusion.

The included studies compared two cohorts of patients: one treated with nCRT followed by surgery and another undergoing direct surgery. Studies included patients submitted to LAR, intersphincteric resection (ISR), or transanal total mesorectal excision (TaTME). Reviews, case reports, editorials, and commentaries were excluded.

Data extracted included: study characteristics (first author name, year); number of patients included in the series; time frame; clinical and demographic characteristics of the patient series; preoperative treatment; surgical approach (open vs laparoscopic); diverting stoma; manometry prior and after surgery and variables of manometry. The manometric variables evaluated in the study were as follows: Maximum Resting Pressure (MaxRP), Maximum Squeeze Pressure (MaxSP), Mean Resting Pressure (MRP), Mean Squeeze Pressure (MSP), Sensory Threshold Volume (STV), and Maximal Tolerable Volume (MTV).

Disagreements among authors regarding the articles’ selection were solved by consensus; if no agreement could be reached, a third senior author (MC) made the decision.

### Assessment of study quality and risk of bias

Two investigators (PM and MIH) independently assessed the methodological quality of the studies using the Newcastle–Ottawa Scale (NOS).^19^ The scoring system covers eight items related to three major domains (selection of exposed and non-exposed cohorts, comparability and outcome assessment).

### Certainty assessment of evidence

Two authors (PM and MIH) conducted an independent assessment of the evidence. The GRADE guidelines were used to rate the quality of evidence.^23^ Study limitations, constancy of effect, imprecision, indirectness, and publication bias were considered. The certainty of evidence was evaluated as high, moderate, low, or very low. GRADEpro GDT software was used to prepare the ‘Summary of Findings’.^24^

### Assessment of heterogeneity

To assess heterogeneity, two strategies were used: 1) The Cochrane Chi-squared test (Q-test), the Tau-squared which is the variance of true effects^25^; and 2) graphical exploration with funnel plots.^26^

### Evaluation of effect size

Review Manager (RevMan) Web from the Cochrane Collaboration was utilized to perform the metanalysis.^27^ We selected the Standardized Mean Difference (SMD) as an effective measure for continuous data. For dichotomous variables, odds ratios (OR) with 95% confidence intervals (95% CI) were calculated. Random effects model was used. The threshold of significance was fixed at 0.05. We tested for the interaction between relevant factors and effect size estimates.

## Results

**Figure 1.**
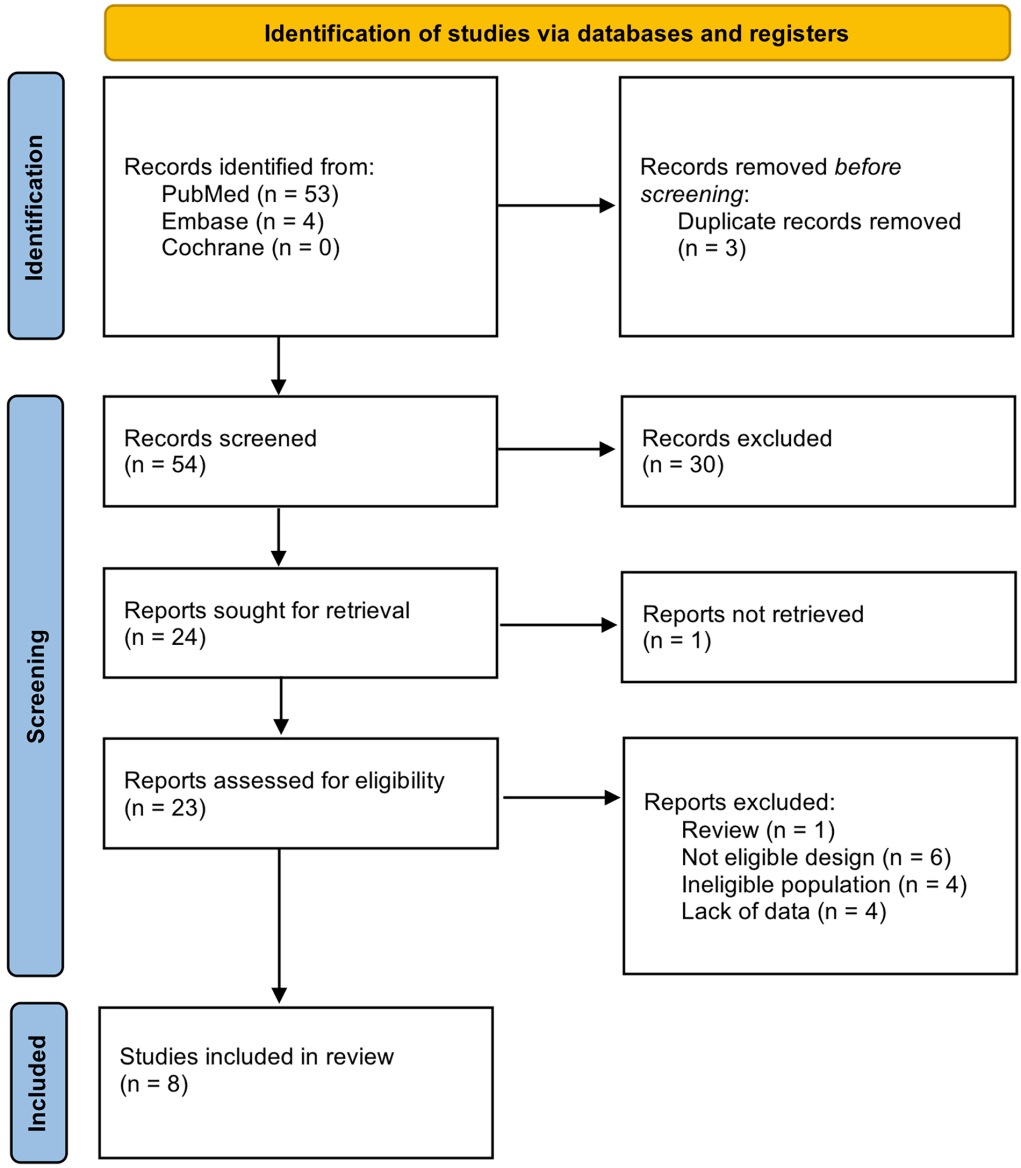
PRISMA statement flow diagram, summary of systematic search and review.

The literature search yielded a total of 57 studies. After screening for duplicates, 54 studies were identified. Among these, 24 studies were found eligible. A total of 16 studies were excluded: one study was a review; one study was not available for downloading; six studies had a design different from that researched; four studies had an ineligible population; four studies lacked manometric data. In total, eight studies were included in the systematic review, of which seven were included in the meta-analysis (Figure 1).

### Systematic review

The included studies were published within the years 2001 to 2024 and encompassed a total of 356 patients. A water-perfused anorectal manometry system was used in six studies,^28-32^ while a high-resolution manometry system was performed in two studies.^33^ One hundred and fifty-five (45.3%) patients were included in the nCRT group. Most patients were male (238 vs. 118). The mean age of the patients included in the study was 63.1 ± 10.4 years. According to the pathological stage, there were 77 (30.7%) patients at stage I, 67 (26.1%) at stage II, 104 (40.6%) at stage III, and 8 (3.1%) at stage IV. van Duijvendijk et al.,^34^ Pi et al.,^35^ and Pietsch et al.^30^ did not report stage classification. Only one study included laparoscopic rectal surgery,^33^ while all the others included open surgery. A diverting ileostomy/colostomy was performed in 265 (74.4%) patients. Tables 1 and 2 show the characteristics of all included studies.

**Table 1.** Characteristics of the included studies.

Author	Year	Title	Journal	Country	Study Type	Indication	Comparison
Gervaz et al.^28^	2001	Quantitative Short-term Study of Anal Sphincter Function After Chemoradiation for Rectal Cancer	Archives of Surgery	USA	CCS	Low and mid rectal adenocarcinoma	nCRT vs US
van Duijvendijk et al.^34^	2002	Prospective Evaluation of Anorectal Function After Total Mesorectal Excision for Rectal Carcinoma with or without Preoperative Radiotherapy	The American Journal of Gastroenterology	Netherlands	RCS	Rectal adenocarcinoma	nCRT vs US
Amman et al.^29^	2003	Impact of Neoadjuvant Chemoradiation on Anal Sphincter Function in Patients with Carcinoma of the Midrectum and Low Rectum	Archives of Surgery	Austria	PCS	Low and mid rectal adenocarcinoma	nCRT vs US
Pietsch et al.^30^	2007	Effect of neoadjuvant chemoradiation on postoperative fecal continence and anal sphincter function in rectal cancer patients	International Journal of Colorectal Disease	Germany	PCS	Low and mid rectal adenocarcinoma	nCRT vs US
Schuld et al.^32^	2010	Reduced neorectal capacitance is a more important factor for impaired defecatory function after rectal resection than the anal sphincter pressure	Colorectal Disease	Germany	RCS	Low and mid rectal adenocarcinoma	nCRT vs TCP vs End to end anastomosis
Canda et al.^31^	2010	Effects of preoperative chemoradiotherapy on anal sphincter functions and quality of life in rectal cancer patients	International Journal of Colorectal Disease	Turkey	PCS	Rectal adenocarcinoma	nCRT vs US
Ihnat et al.^33^	2018	Anorectal dysfunction after laparoscopic low anterior rectal resection for rectal cancer with and without radiotherapy (manometry study)	Journal of Surgical Oncology	Czech Republic	PCS	Low and mid rectal adenocarcinoma	nCRT vs US
Pi et al.^35^	2022	Anorectal dysfunction in patients with mid-low rectal cancer after surgery: A pilot study with three-dimensional high-resolution manometry	World Journal of Clinical Cases	China	RCS	Low and mid rectal adenocarcinoma	nCRT vs US

CCS, case-control study; nCRT, neoadjuvant chemoradiotherapy; RCS, retrospective cohort study; PCS, prospective cohort study; TCP, transverse coloplasty; US, upfront surgery.

**Table 2.** Baseline demographics of the included studies.

Author	Year	Sample size (number)	Age (years)	Gender (Male: Female)	Neo-adjuvant chemotherapy	Type of ARM	Timing of ARM after surgery
Gervaz et al.^28^	2001	nCRT: 18 US: 23	nCRT: 68 ± 9 US: 66 ± 12	nCRT: 16:3 US: 16:7	Long course	Water perfused manometry system	NR
van Duijvendijk et al.^34^	2002	nCRT: 14 US: 20	nCRT: 69 (38-85) US: 65 (38-83)	nCRT: 9:5 US: 16:4	Short course	Water perfused manometry system	1 year
Amman et al.^29^	2003	nCRT: 28 US: 22	nCRT: 59 ± 10 US: 63 ± 14	nCRT: 21:7 US: 11:11	Long course	Water perfused manometry system	Median 384 (IQR, 149-405) days
Pietsch et al.^30^	2007	nCRT: 12 US: 27	nCRT: 59.8 ± 11.9 US: 61.9 ± 10.6	nCRT: 8:4 US: 16:11	Long course	Water perfused manometry system	3-6 months (after closure of ileostomy)
Schuld et al.^32^	2010	45	65.6 ± 1.6	33:12	NR	Water perfused manometry system	21.6 ± 1.4 months
Canda et al.^31^	2010	nCRT: 31 US: 26	nCRT: 59 (38-76) US: 65 (41-85)	nCRT: 18:13 US: 19:7	Long course	Water perfused manometry system	Median 417 (149-477) days
Ihnat et al. ^33^	2018	nCRT: 38 US: 27	nCRT: 62.1 ± 10.8 US: 66.1 ± 8.5	nCRT: 25:13 US: 16:11	Long course	ManoScan 3D HR-ARM	1 year
Pi et al.^35^	2022	nCRT: 18 US: 6	57.7 ± 10.4	14:10	NR	ManoScan 3D HR-ARM	3-6 months

nCRT, neoadjuvant chemoradiotherapy; US, Upfront surgery; ARM, anorectal manometry; HR-ARM, high resolution ARM; NR, not recorded.

Ihnat et al. included 65 patients.^33^ A significantly lower RP was reported in the group of patients in the nCRT group compared to LAR upfront (p<0.001). Interestingly, the values of SP were similar between the two groups (p=0.254). Rectal compliance and volumes describing rectal sensitivity were significantly lower in the irradiated patients (p<0.001).

As commented by Gervaz et al.,^28^ postoperative RP was significantly lower in the nCRT group (p=0.03). However, SP was similar between the two groups (p=0.69). Also, STV and MTV were not different between the two groups (p=0.72 and p=0.65, respectively).

Schuld et al.^32^ reported a worse defecatory function, in terms of fecal incontinence score, in the nCRT group compared to the LAR upfront group (p<0.05). However, the only different value at the manometry examination, between the two groups, was the MTV of the neorectum, which was higher in patients who underwent nCRT versus a transverse coloplasty and an end-to-end anastomosis (p<0.05).

Canda et al.^31^ reported that patients treated with nCRT had a significantly lower maximum SP (p<0.05). All other post-operative parameters were similar between the two groups. In addition, the threshold for first sensation and maximum tolerable volume measurements and disappearance of rectoanal inhibitory reflex (RAIR) were not significantly different between the groups in all the considered periods.

Pietsch et al.^30^ reported no statistical difference between patients with or without nCRT, however fecal continence, measured through ARM and analysis of symptoms, was significantly impaired in both groups after surgery.

Amman et al.^29^ found that LAR upfront patients did not experience differences in manometric values between pre-operative and post-operative time, while nCRT patients had a lower post-operative mean RP and MTV (p<0.05).

van Duijvendijk et al.^34^ demonstrated that functional outcome was poor in both nCRT and upfront surgery, with a significantly higher defecation frequency after nCRT as compared with TME. There were no significant differences in anal sphincter function or rectal sensitivity to pressure-controlled distention between the two treatments.

Pi et al.^35^ found that surgery significantly reduced the length of the high-pressure zone of the anal sphincter, as well as the mean resting and maximal squeeze pressures of the anus in all patients (p<0.05). This effect was most pronounced in the neoadjuvant therapy group (n = 18).

### Meta-analysis

**Figure 2.**
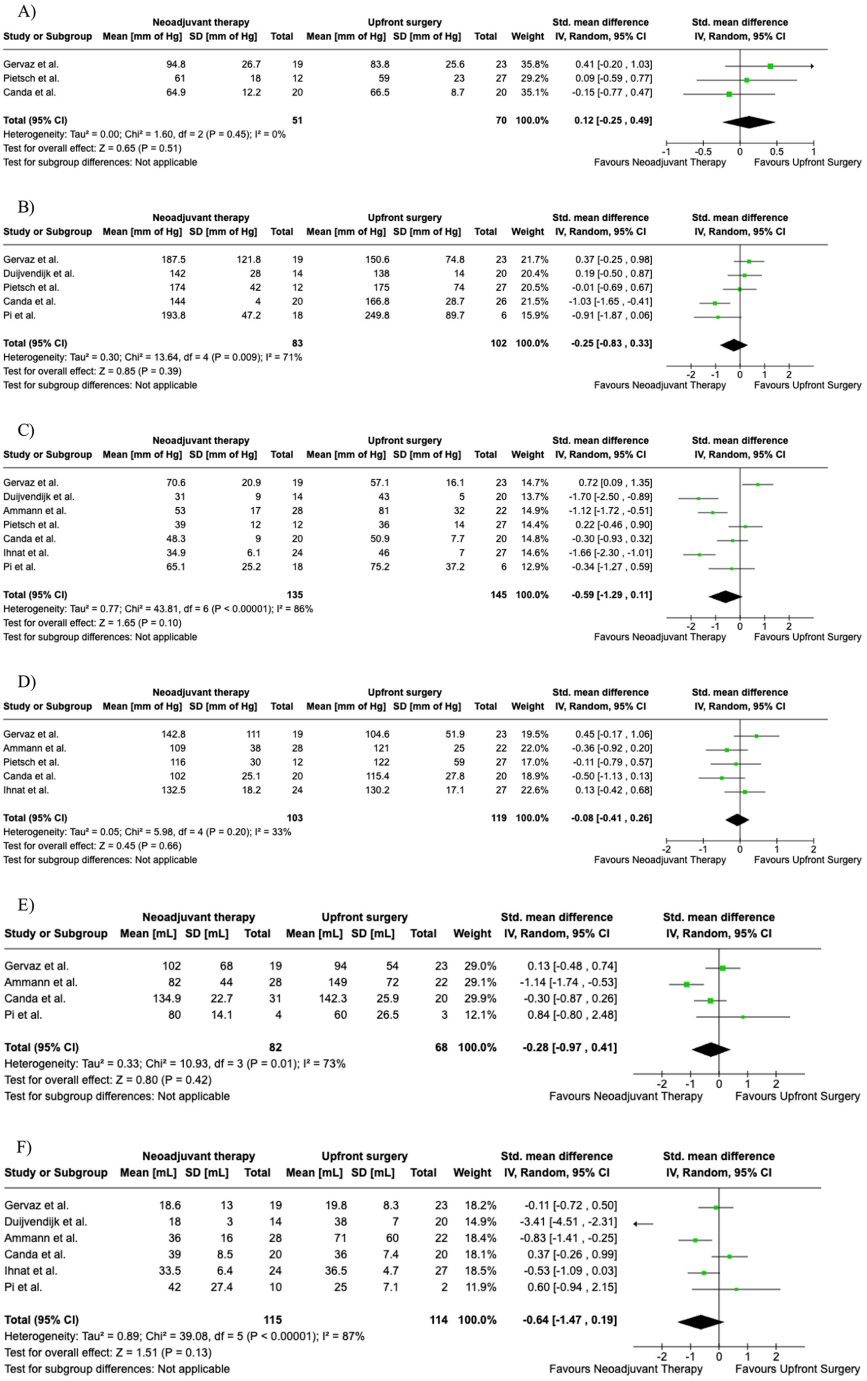
Random funnel plot for Maximum Resting Pressure (MaxRP, A), Maximum Squeeze Pressure (MaxSP, B), Mean Resting Pressure (MRP, C), Mean Squeeze Pressure (MSP, D), Maximal Tolerable Volume (MTV, E), and Sensory Threshold Volume (STV, F).

Three studies reported Max RP (Figure 2A). It was reported in 51 out of 155 patients in the nCRT group and 70 out of 187 patients in the upfront surgery group. There was no difference between the two groups (SMD = 0.12; 95% CI [-0.25, 0.49], p = 0.51). There was low heterogeneity among the studies (I² = 0).

Five studies reported Max SP (Figure 2B), which included 83 out of 155 patients in the nCRT group compared to 102 out of 187 patients in the upfront surgery group. There was no difference between the two groups (SMD = -0.25; 95% CI [-0.83, 0.33], p = 0.39). There was high heterogeneity among the studies (I² = 71%).

Seven studies reported MRP (Figure 2C). which included 135 out of 155 patients in the nCRT group compared to 145 out of 187 patients in the upfront surgery group. There was no difference between the two groups (SMD = -0.59; 95% CI [-1.29, 0.11], p = 0.10). There was high heterogeneity among the studies (I² = 86%).

Five studies reported MSP (Figure 2D). It was reported in 103 out of 155 patients in the nCRT group and 119 out of 187 patients in the upfront surgery group. There was no difference between the two groups (SMD = -0.08; 95% CI [-0.41,0.26], p = 0.66). There was moderate heterogeneity among the studies (I² = 33%)

Four studies reported MTV (Figure 2E). It included 82 out of 155 patients in the nCRT group and 68 out of 187 patients in the upfront surgery group. There was no difference between the groups (SMD = -0.28; 95% CI [-0.97, 0.41], p = 0.42). There was high heterogeneity among the studies (I² = 73%).

Six studies reported STV (Figure 2F), which included 115 out of 155 patients in the nCRT group compared to 114 out of 187 patients in the upfront surgery group. There was no difference between the two groups (SMD = -0.64; 95% CI [-1.47, 0.19], p = 0.13). There was high heterogeneity among the studies (I² = 87%).

### Quality assessment of the included studies

The NOS scale is reported in Table 3 and a summary of the evidence is presented in Table 4.

**Table 3.** Newcastle–Ottawa Scale quality evaluation table.

Study	Selection	Comparability	Outcomes	Total
	1 2 3 4	1 2	1 2 3	
Gervaz et al., 2001^28^	★ ★ ★	★	★ ★	6
van Duijvendijk et al., 2002^34^	★ ★ ★	★ ★	★ ★	7
Amman et al., 2003^29^	★ ★	★	★ ★	5
Pietsch et al., 2007^30^	★ ★	★	★ ★ ★	6
Schuld et al., 2010^32^	★ ★		★ ★	4
Canda et al., 2010^31^	★ ★ ★	★	★ ★ ★	7
Inhat et al., 2017^32^	★ ★ ★ ★	★	★ ★ ★	8
Pi et al., 2022^35^	★ ★ ★ ★	★ ★	★ ★	8

Each study is judged on a “star system” based on the selection of the study groups the ascertainment of the outcome of interest. Each study can earn a maximum of nine stars.

**Table 4.** Summary of findings.

Outcomes	No of participants (studies) Follow-up	Certainty of the evidence (GRADE)	Relative effect (95% CI)	Anticipated absolute effects	
				Risk with upfront surgery	Risk difference with neoadjuvant therapy
Maximum Resting Pressure	121 (3 non-randomized studies)	⨁⨁⨁⨁ High	-	The mean maximum Resting Pressure was **0**	MD **0.73 higher** (5.14 lower to 6.61 higher)
Maximum Squeeze Pressure	185 (5 non-randomized studies)	⨁⨁⨁⨁ High	-	The mean maximum Squeeze Pressure was **0**	MD **7.42 lower** (27.96 lower to 13.12 higher)
Maximal Tolerable Volume	150 (4 non-randomized studies)	⨁⨁◯◯ Low	-	The mean maximal Tolerable Volume was **0**	MD **11.42 lower** (42.87 lower to 20.03 higher)
Mean Resting Pressure	280 (7 non-randomized studies)	⨁⨁⨁⨁ High	-	The mean Resting Pressure was **0**	MD **6 lower** (12.97 lower to 0.97 higher)
Mean Squeeze Pressure	222 (5 non-randomized studies)	⨁⨁⨁⨁ High	-	The mean mean Squeeze Pressure was **0**	MD **4.29 lower** (14.45 lower to 5.87 higher)
Sensory Threshold Volume	229 (6 non-randomized studies)	⨁⨁⨁⨁ High	-	The mean Sensory Threshold Volume was **0**	MD **5.41 lower** (15.42 lower to 4.6 higher)

**The risk in the intervention group** (and its 95% confidence interval) is based on the assumed risk in the comparison group and the **relative effect** of the intervention (and its 95% CI).

**CI:** confidence interval; MD: mean difference.

**GRADE Working Group grades of evidence**.

**High certainty:** we are very confident that the true effect lies close to that of the estimate of the effect.

**Moderate certainty:** we are moderately confident in the effect estimate: the true effect is likely to be close to the estimate of the effect, but there is a possibility that it is substantially different.

**Low certainty:** our confidence in the effect estimate is limited: the true effect may be substantially different from the estimate of the effect.

**Very low certainty:** we have very little confidence in the effect estimate: the true effect is likely to be substantially different from the estimate of effect.

## Discussion

This systematic review and metanalysis failed to demonstrate any statistically significant difference between nCRT and upfront surgery regarding standardized mean difference of mean RP, mean and maximum SP, STV, and MTV. The standardized mean difference of maximum RP favored upfront surgery but with no statistical difference. This contradicts with the literature,^9,10,31^ where, however, an objective evaluation of nCRT effects on the anal function has not yet been fully characterized. Risk factors for developing LARS are usually considered: nCRT,^10,36^ low anastomotic site,^37^ age above 70 years, female gender,^4,38^ and postoperative complications such as anastomotic leak.^39^

The surgical factors involved in LARS development remain unclear, however many different hypotheses have been suggested. First, fecal and/or gas incontinence seems to be related to the direct structural damage, caused by the intersphincteric resection technique itself, and indirectly by the introduction of the stapler into the anus.^8^ Second, the effects on the nervous system and sphincter complex due to direct damage to the nervous plexus have been advocated as one of the main factors inducing LARS.^40^ Thirdly, it has been suggested that the level of continence could be also affected by the decreased compliance of the neo-rectum reservoir.^37^ However, there is still limited data evaluating the sphincter pathophysiology and damage evaluation following surgical intervention, nCRT dose, protocol and timing.

The role of ARM is to objectively evaluate the anal sphincter function and rectal capacity and to record parameters like RP, maximum SP, RAIR, rectal capacity, and compliance. The function of ARM has not been suggested for the diagnosis of LARS, but as a tool to monitor the efficacy of the therapy in improving continence.^41^

The effects of LAR on continence and compliance of the rectum could be measured through STV and MTV. Both values are expected to decrease after surgery due to the lower capacity of the neo-rectum. Moreover, nCRT could also affect rectal compliance by causing radiation-induced fibrosis.^42^ The high frequency of patients’ symptoms like fecal urgency and increased frequency of bowel movements are complained after pelvic irradiation not only for RC but also for prostate or cervical cancer,^43^ therefore highlighting the role of nCRT as a risk factor for LARS. Expectedly, the results of this study demonstrated lower STV (SMD: -0.64; p=0.13) and MTV (SMD=-0.28; p=0.42) in the nCRT group compared to the upfront surgery group; however, the difference is not statistically significant.

RP and SP are other manometric parameters related to the injury of the anal sphincter, through direct or nervous damage. RP is mainly produced by the tonic contractions of the internal anal sphincter, through parasympathetic fibers of the pelvic splanchnic nerves. Postoperative anal dysfunction was reported mostly in patients treated with nCRT compared to adjuvant radiotherapy, as suggested by the pathological findings,^35-43^ of neural degeneration following nCRT in RC patients from Koushi et al.^43^ Also, the mechanical damage to the internal anal sphincter caused by the stapler introduction could cause a significant decrease in RP as reported by Farouk et al.^8^

The present study shows that RP was more negatively influenced by nCRT compared to upfront surgery (SMD=- 0.59, p=0.10). On the other hand, SP is not expected to be influenced by surgery as it is generated by contraction of the external anal sphincter and the puborectalis muscle, which should be not injured during LAR.^41^ However, the influence of radiotherapy on SP has been demonstrated to lead to different and contrasting outcomes, as reported by a review of Krol et al..^44^ This study reports that SP seems to be negatively influenced by nCRT compared to upfront surgery, even if not reaching a statistical significance (SMD= -0.08, p=0.66).

This study has several limitations related to the nature of meta-analysis. First, this study was performed through rigorous inclusion criteria with a consequent selection of “high quality” studies. However, a selection bias could always occur and could have affected the results of the study. Second, by including only published studies, which usually report significant results (publication bias), this study may have overestimated the actual magnitude of nCRT on anorectal function. Third, there was moderate to substantial heterogeneity among the studies on all four endpoints (SP, RP, STV, and MTV); this could have affected the results. Fourth, ARM is an operator-dependent procedure that requires highly trained professionals to acquire high-quality data. Fifth, ARM is also platform-dependent with differences in results arising according to the instrument used;^45-48^ this could have affected the results of the study. Sixth, quality of the included studies is limited.

Therefore, it can be suggested that the anorectal functional evaluation should be performed by highly specialized teams able to evaluate the patient’s functional outcomes in a more comprehensive way using QoL questionnaires, instrumental assessments (i.e., ARM), together with electromyography, defecography/magnetic resonance, and pelvic floor ultrasound.

Further studies on homogenous populations treated by experienced teams with standardized protocols and platforms are required to further evaluate the effects of nCRT on anorectal function. The strength of the present study is to provide a review and meta-analysis on anorectal function specifically focused on nCRT effects objectively characterized using ARM.

In conclusion, this study objectively evaluates and confirms the negative effect of nCRT on the anorectal function after LARFurther, well-designed trials using of standardized platforms and procedures are needed to confirm the results of this meta-analysis. The use of the 3-D anorectal high-resolution manometry could be of great help in better understanding the baseline function of the anorectum and further evaluating surgical and chemoradiation effects on it.
